# Sex-specific correlates of *Schistosoma haematobium* infection in a rural Zambian setting: A pilot study focusing on immune and hematological factors

**DOI:** 10.1371/journal.pgph.0005692

**Published:** 2026-11-04

**Authors:** David Chisompola, Reggison Phiri, Emmanuel L. Luwaya, Martin Chakulya, Lukundo Siame, Bislom C. Mweene, Benson M. Hamooya, Joseph M. Chalwe, Joreen P. Povia, Sepiso K. Masenga

**Affiliations:** 1 HAND Research Group, School of Medicine and Health Sciences, Mulungushi University, Livingstone, Zambia; 2 Department of Pathology, Livingstone Center for Prevention and Translational Science, Livingstone, Zambia; 3 Nuclear Medicine Research Infrastructure (NuMeRI), Capital Park, Pretoria, South Africa; 4 Department of Nuclear Medicine, University of Pretoria & Steve Biko Academic Hospital, Pretoria, South Africa; 5 Department of Economics, Livingstone Center for Prevention and Translational Science, Livingstone, Zambia; PLOS: Public Library of Science, UNITED STATES OF AMERICA

## Abstract

This study aimed to identify and compare the sex-specific immune, hematological, and environmental factors associated with *Schistosoma haematobium* infection in a rural Zambian setting. This community-based cross-sectional study enrolled participants from rural Zambia. *Schistosoma haematobium* infection was diagnosed microscopically from urine. Blood samples were analyzed for complete blood counts and cytokines via ELISA. Data were analyzed using sex-stratified logistic regression models in StatCrunch, with multivariable models restricted to a limited number of predictors based on events-per-variable considerations to improve stability. The analysis included 51 male and 92 female participants. In the multivariable model, for males, only access to clean water remained independently associated with schistosomiasis infection (aOR = 0.03, 95% CI: 0.00–0.34, p = 0.0040). For females, the final model identified access to clean water (aOR = 0.11, 95% CI: 0.01–0.93, p = 0.0440), reporting any symptoms (aOR = 19.2, 95% CI: 3.17–116.4, p = 0.0010), elevated IL-4 (aOR = 1.01, 95% CI: 1.00–1.02, p < 0.0001), and lower hemoglobin (aOR = 0.48, 95% CI: 0.28–0.83, p = 0.0080) as independent correlates of infection. The correlates of schistosomiasis infection appear to differ by sex. While environmental exposure is a key driver for males, the female profile reflects a combination of environmental, immunological, and hematological factors. These findings highlight the importance of considering sex-specific patterns in schistosomiasis research; however, given the cross-sectional design and limited sample size, the results should be interpreted with caution and confirmed in larger, longitudinal studies.

## Introduction

Schistosomiasis remains one of the most prevalent and devastating neglected tropical diseases (NTDs), with a global burden disproportionately shouldered by sub-Saharan Africa [1]. Of the human-pathogenic species, *Schistosoma haematobium* is responsible for urogenital schistosomiasis, a chronic infection estimated to affect over 110 million people, with the vast majority of cases concentrated in low income countries [2].

The pathology of *S. haematobium* arises primarily from parasite eggs becoming trapped in the walls of the bladder, ureters, and genital tissues. This triggers a granulomatous inflammatory response that can cause hematuria, fibrosis, urinary obstruction, and an increased risk of bladder cancer [3]. The host counters egg deposition with a T-helper 2 (Th2) immune response, characterized by the production of interleukin (IL)-4, IL-5, IL-13, and IgE [4,5]. While this Th2 response is essential for containing the infection, it is concurrently regulated by immunomodulatory pathways, such as regulatory T cells (Tregs) and IL-10, to prevent excessive tissue damage [6]. The important balance between these pro-inflammatory and regulatory forces ultimately determines infection outcomes, ranging from resistance to reinfection to the development of severe morbidity [7].

Studies across *Schistosoma* species have revealed both conserved and species-specific immune features. Research on *S. mansoni* has established the fundamental paradigm that egg deposition drives a robust Th2-polarized response, with IL-4 playing a central role in granuloma formation and fibrosis [8]. Murine models of *S. mansoni* infection have demonstrated that signal transducer and activator of transcription 6 (STAT6) and IL-4 receptor alpha signaling are essential for granuloma development, as their ablation markedly reduces pathology [9]. More recently, investigations into *S. japonicum* have shown that glucocorticoid receptor activation can suppress Th2 responses by downregulating the transcription factors Gata3 and Pparg, offering potential therapeutic insights applicable across schistosome species [10].

The cytokine milieu in schistosomiasis extends beyond the classic Th2 axis. Tumor necrosis factor (TNF) has been implicated in both limiting liver pathology and promoting adult parasite survival in *S. mansoni* infections, suggesting a complex host-parasite co-evolution where inflammatory mediators serve dual roles [11]. Studies of circulating cytokines in *S. haematobium*-endemic populations in Zimbabwe have revealed that egg-positive individuals exhibit significantly higher levels of IL-2, IFN-γ, and IL-23, while egg-negative individuals demonstrate elevated IL-10, IL-4, IL-13, and IL-21 [12]. This mixed Th1/Th2/regulatory profile in patent infection, contrasting with a Th2-dominated suite in egg-negative individuals, underscores the dynamic nature of the host response and the importance of immunoregulation in determining infection outcomes.

Moreover, hematological alterations are also a recognized feature of chronic schistosomiasis. Anemia in *S. mansoni* and *S. haematobium* has been attributed to chronic inflammation, iron sequestration mediated by hepcidin, occult blood loss, and bone marrow suppression driven by inflammatory cytokines [13,14]. However, most immunopathological studies of human schistosomiasis focus on single-sex cohorts or fail to stratify by biological sex, despite mounting evidence that sex hormones modulate cytokine production, antibody responses, and eosinophil activity.

Biological sex is an important modifier of infection outcomes. Sex hormones directly influence immune function, and immunological dimorphism may explain sex-biased infection and pathology beyond just behavioral differences [15–20]. While murine models of *S. mansoni* show females mount stronger immune responses [21], sex-specific immune and hematological correlates in *S. haematobium* infection remain a significant knowledge gap. Furthermore, a growing body of evidence has established a robust association between female genital schistosomiasis (FGS) and heightened susceptibility to HIV, underscoring the infection’s role as a critical, yet often overlooked, co-morbidity [22–24].

In schistosomiasis, some studies have reported higher prevalence and infection intensities in males, often attributed to gender-based differences in water-contact behavior [25]. However, a purely behavioral explanation is insufficient, as emerging evidence points to intrinsic biological factors that may render one sex more susceptible to infection or pathology. Most immunopathological studies of schistosomiasis either do not stratify their analyses by sex or lack the power to detect sex-specific effects [26].

This omission is essential, as sex-specific biology may influence reinfection rates, pathology, and the success of public health interventions like mass drug administration (MDA) with praziquantel [27–29]. This study aims to identify the sex-specific immune and hematological correlates of S. haematobium infection in a rural Zambian community. We hypothesize that distinct profiles between males and females are linked to differences in infection intensity and morbidity, knowledge essential for developing more effective, stratified control strategies.

## Methodology

### Study design

A community-based cross-sectional study was carried out in the rural communities of Machile, Sichili, and Mulobezi within the Mulobezi District of Western Zambia [30]. A total of 143 participants (92 females, 51 males) across pediatric and adult age groups were enrolled. This sex distribution reflects the demographic and practical realities of the study setting, where females were more frequently available at home during daytime recruitment visits. Enrollment was voluntary and no potential participants were excluded based on sex; the imbalance arose naturally from community availability and willingness to participate. While this provided adequate power to explore female-specific correlates, a priority given the focus on immunohematological manifestations like anemia, we acknowledge that the smaller male subgroup may limit the detection of more subtle correlates in men.

### Eligibility criteria

The study enrolled residents of the district, aged 5–55 years, who had resided in the area for a minimum of three months. All participants provided informed consent; for children under 16 years, assent was obtained from the child alongside consent from a parent or guardian. Pregnant individuals and those with serious comorbidities, such as advanced HIV or autoimmune diseases, were excluded.

### Data and sample collection

Participants were recruited between 2^nd^ January 2024 and 1^st^ June 2024. Participants underwent a structured interview to gather demographic and behavioral data.

**Assessment of Signs and Symptoms**: Clinical signs and symptoms of urogenital schistosomiasis were assessed through age-appropriate, standardized approaches conducted by trained study clinicians (clinical officers and medical doctors). Given that the study population included both children and adults (age range 5–55 years, median 15 years), special consideration was given to adolescent participants to ensure comfort and appropriate communication during assessments.

**Symptom Assessment**: All participants or their caregivers (for younger children) underwent a structured interview administered by trained research nurses in the local language (Tonga/Lozi). The interview inquired about the presence of classic urogenital schistosomiasis symptoms in the preceding four weeks, including: visible hematuria (blood in urine), dysuria (painful urination), frequent urination, and suprapubic discomfort or report of these symptoms by a study participant. For adolescent female participants (≥12 years), age-appropriate questions were carefully framed to inquire about potential symptoms of female genital schistosomiasis, including vaginal discharge, genital itching, and contact bleeding, with a parent or guardian present if the participant and guardian consented. For younger children (<12 years), symptom history was obtained primarily from the parent or guardian.

**Clinical Examination**: Each participant received a brief physical examination by a study clinician in the presence of a same-gender chaperone. For all participants, this included assessment for visible hematuria and suprapubic tenderness on palpation. For adolescent participants, examinations were conducted with strict attention to privacy and comfort.

**Definition of Any Signs/Symptoms**: For the purpose of analysis, participants were classified as having ‘any signs/symptoms’ if they reported at least one symptom during the age-appropriate interview OR had at least one clinical sign identified on examination that was attributable to urogenital schistosomiasis. This composite variable captured both symptomatic presentations (e.g., dysuria reported by an adolescent) and objective findings (e.g., visible hematuria observed by the clinician).

All clinical assessments were conducted by trained study clinicians, and standardized training was provided to ensure consistency in symptom inquiry and examination techniques across the study team, with additional training on age-appropriate communication with adolescent participants.

**Protective Measures:** Participants were asked about their use of personal protective behaviors when engaging in activities that involved contact with surface water (rivers, streams, dams). Based on preliminary community engagement and known local practices, the following specific measures were inquired about:

1. Footwear use: Wearing closed shoes, boots, or sandals when entering water
2. Glove use: Wearing protective gloves when washing clothes or dishes in river water
3. Water handling practices: Allowing river water to settle before use, or using containers to collect water without full body immersion
4. Avoidance behaviors: Deliberately avoiding water contact during peak cercarial shedding times (mid-day to early afternoon) when community knowledge indicates higher risk

Participants were asked to report their typical practices and were categorized based on their response to a single question: “How often do you use any protective measures (such as those we discussed) when you come into contact with river or stream water?” Response options were: “Always” (consistently use one or more measures), “Sometimes” (occasionally use measures), or “Never” (do not use any protective measures). This composite variable was used in the analysis as “protective measures.”

**Water Contact Activities**: Engagement in activities with contaminated water was defined as the self-reported frequency of contact with natural freshwater bodies (rivers, streams, and dams) known to be potential transmission sites for schistosomiasis in the study area. Participants were asked to indicate how often they engaged in such activities (daily, weekly, rarely, or never).

Biological samples collected included two venous blood samples and a single midstream urine sample. The urine specimens were transported under cool conditions to a local hospital for immediate microscopic analysis. Blood samples were processed for a complete blood count at a certified clinical laboratory, while separated serum was shipped to a specialized research facility for cytokine quantification.

### Laboratory procedures

Diagnosis of *S. haematobium* infection was confirmed through microscopic identification of ova in centrifuged urine sediment [31]. A 10 mL of well-mixed urine was centrifuged at 1500–2000 rpm for 5 minutes. The supernatant was discarded, and the entire sediment was transferred to a glass slide and examined systematically under light microscopy at X10 and X40 magnification for the presence of characteristic terminal-spined *S. haematobium* eggs. All examinations were performed independently by two trained laboratory technologists; in cases of disagreement, a third technologist served as the tie breaker.

Plasma concentrations of the cytokines IL-4 and IL-10 were determined using commercially available, validated enzyme-linked immunosorbent assay (ELISA) kits Elabscience (Elabscience Biotechnology Inc., Houston, TX, USA; IL-4: Catalog. No. E-EL-H0101; IL-10: Catalog. No. E-EL-R0016), adhering strictly to the manufacturer’s protocols.

### Statistical analysis

Data were analyzed using StatCrunch software. Descriptive statistics summarized participant characteristics. The Mann-Whitney U test was employed for non-normally distributed continuous variables, and the Chi-square test assessed associations between categorical variables. To identify infection correlates, sex-stratified univariate and multivariable logistic regression models were constructed, with statistical significance defined as a p-value of less than 0.05.

Given the limited number of infection events in the sex-stratified analyses (e.g., 38 events in female’s vs 16 events in males), multivariable logistic regression models were restricted to a parsimonious set of predictors to reduce overfitting, guided by events-per-variable considerations. Variables with p < 0.05 in the unadjusted logistic regression analysis were selected for inclusion in the multivariable logistic regression model. The same set of variables were included in the adjusted models for males and females to facilitate direct comparison between the two groups. This approach was adopted to minimize the risk of overadjustment and model instability while maintaining consistency in the variables included across sex-specific models.

### Ethical considerations

The study protocol received full approval from Mulungushi University School of Medicine and Health Sciences Ethics Review Committee (Ref. No.: SMHS-MU4-2023-031) on 26^th^ December 2023. Prior to enrollment, all participants provided written informed consent/assent after a detailed explanation of the study in their local language, ensuring voluntary participation and confidentiality. Data were anonymized to protect confidentiality. All data collected and analyzed were de-identified to ensure complete confidentiality. No information leading to identification of patients during and after analysis was abstracted and entered in the data collection form.

We used the Strengthening the Reporting of Observational Studies in Epidemiology (STROBE) for reporting (S1 STROBE Checklist) and the data supporting the results is available as supplementary file, S1 Data.

## Results

### Characteristic of the study population

Baseline characteristics of the 143 participants, stratified by biological sex. Data are shown as median (IQR) (Table 2) or n (%) (Table 1). Statistical comparisons revealed that the male (n = 51) and female (n = 92) cohorts were well-matched, showing no significant differences in key exposure and clinical parameters at baseline analysis (p > 0.05) (Table 1).

**Table 1:** Comparison of participant characteristics by sex.

Variable	Median (IQR)/Frequency (%)	Male = 51 (35.7)	Female = 92 (64.3)	P value
**Access to clean water**				
Yes	55 (38,5)	22 (43.1)	33 (35.8)	0.3922
No	88 (61.5)	29 (56.9)	59 (64.1)	
**Source of drinking water**				
Tap	55 (38.5)	22 (43.1)	32 (34.8)	0. 3236
River	88(61.5)	29 (56.9)	59 (64.1)	
**Signs/Symptoms**				
Yes	64 (44.8)	25 (49.0)	39 (42.4)	0.4451
No	79 (55.2)	26 (51.0)	53 (57.6)	
**Schistosomiasis**				
Yes	56 (39.2)	18 (35.3)	38 (41.3)	0.4806
No	87 (60.8)	33 (64.7)	54 (58.7)	
**Have you ever been diagnosed**				
Yes	25 (17.5)	10 (19.6)	15 (16.3)	0.6183
No	118 (82.5)	41 (80.4)	77 (83.7)	
**Ever been treated for other parasitic**				
Yes	138 (96.5)	2 (3.9)	8 (8.7)	0.4950
No	5 (3.5)	49 (96.1)	84 (91.3)	
**On treatment for other condition**				
Yes	16 (11.2)	4 (7.8)	12 (13.0)	0.4167
No	127 (88.8)	47 (92.2)	80 (87.0)	
**Education**				
Not Schooled	22 (15.4)	7 (13.7)	15 (16.3)	0.6822
Primary	102 (71.3)	41 (80.4)	61 (66.3)	
Secondary	18 (12.6)	2 (3.9)	16 (17.4)	
Tertiary	1 (0.7)	1 (2.0)	0 (0)	
**Protective measures**				
Always	7 (4.9)	4 (7.8)	3 (3.3)	0.2238
Sometime	98 (68.5)	27 (52.9)	71 (77.2)	
Never	38 (26.6)	20 (39.2)	18 (19.5)	
**Engage in activities with contaminated** water				
Daily	123 (86)	44 (86.2)	79 (85.9)	0.5188
Weekly	13 (9.1)	6 (11.8)	7 (7.6)	
Monthly	0	0 (0)	0 (0)	
Rarely	5 (3.5)	1 (2.0)	4 (4.3)	
Never	2 (1.4)	0 (0)	2 (2.2)	

Comparisons between male and female participants were performed using the Chi-square test. A p-value <0.05 was considered statistically significant.

### Baseline demographic, inflammatory, and hematological characteristics

Statistical comparisons indicated that male and female cohorts were generally comparable at baseline analysis, with no statistically significant differences in age, cytokine levels, or most hematological parameters. However, significant differences were observed in mean corpuscular volume (p = 0.0454), mean corpuscular hemoglobin (p < 0.0001), and mean corpuscular hemoglobin concentration (p = 0.0029) (Table 2).

**Table 2:** Comparison of demographic, inflammatory, and hematological parameters between male and female participants.

Variable	Median (IQR)	Male = 51 Median (IQR)	Female = 92 Median (IQR)	P value
Age (yrs)	15 (9,30)	13 (8,20)	16 (10.5,30)	0.0632
IL 4 (pg/mL)	57.1 (29.4, 312.5)	47.6 (25.5,259.3)	66.7 (31.97,316.55)	0.2979
IL 10 (pg/mL)	26.7 (19.4, 50.9)	33.53 (20.23,50.58)	26.27 (19.16,51.22)	0.7939
Eosinophil	0.12 (0.07, 0.4)	0.11 (0.07,0.28)	0.12 (0.07,0.49)	0.3394
RBC (x10^12^/L)	4.51 (4.12, 5)	4.51 (4.12,5.1)	4.52 (4.115,4.89)	0.7013
Hb (g/dL)	12 (10.9, 13.2)	12 (11.1, 13.3)	11.95 (10.7, 13)	0.8993
MCV (fL)	87 (81.5, 92)	88 (81.5, 92)	86.55 (81.5, 92)	**0.0454**
MCH (pg)	27.1 (24.4, 28.5)	27.1 (24.8, 28.3)	26.9 (24.2, 28.6)	**<0.0001**
MCHC (g/dL)	30.1 (29.2, 30.9)	30.2 (29.4, 30.9)	30.1 (29.15, 30.9)	**0.0029**
Neutrophil (x10^9^/L)	1.24 (1.01, 1.96)	1.17 (0.93,1.88)	1.27 (1.01, 2.08)	0.5551
Lymphocyte (x10^9^/L)	2.4 (1.68, 2.94)	2.1 (1.68, 2.7)	2.45 (1.72, 3.4)	0.0601
Monocyte (x10^9^/L)	0.24 (0.14, 0.62)	0.24 (0.14, 0.6)	0.24 (0.14, 0.63)	0.6128
Platelet (x10^9^/L)	244 (190, 285)	245 (180, 295)	244 (191.5, 284.5)	0.9027
PLR	104.9 (71.6,140.4)	108.7 (80.6, 142.3)	97.6 (63.1, 138.5)	0.2388
LMR	9.2 (4.2, 13.9)	8.7 (3.9, 13.9)	9.3 (5.0, 12.8)	0.7535
NLR	0.58 (0.42, 0.86)	0.63 (0.48, 0.83)	0.56 (0.38, 0.89)	0.2844

Abbreviations: IQR, interquartile range; IL-4, interleukin-4; IL-10, interleukin-10; RBC, red blood cell; Hb, hemoglobin; MCV, mean corpuscular volume; MCH, mean corpuscular hemoglobin; MCHC, mean corpuscular hemoglobin concentration; PLR, platelet-to-lymphocyte ratio; LMR, lymphocyte-to-monocyte ratio; NLR, neutrophil-to-lymphocyte ratio. Continuous variables are presented as median (IQR). Comparisons between male and female participants were performed using the Mann–Whitney U test. Statistically significant p-values (p < 0.05) are shown in bold.

### Association in logistic regression

Table 3 presents the univariate analysis, showing the crude association between each variable and schistosomiasis, separately for males and females. For Males: Several factors were significantly associated with schistosomiasis. The strongest protective factor was access to clean water (OR=0.03, 95% CI: 0.00-0.29, p = 0.0019), while the strongest risk factor was reporting any symptoms (OR=53.13, 95% CI: 6.08-464.48, p = 0.0003). Using an unprotected water source was a major risk (OR=29.75, 95% CI: 3.51-252.33, p = 0.0019), as was not using protective measures (OR=3.02, 95% CI: 1.04-8.79, p = 0.0422). A higher lymphocyte count was also a significant risk factor (OR=2.11, 95% CI: 1.04-4.26, p = 0.0378) (Table 3).

**Table 3:** Sex differences in correlates of schistosomiasis in univariate analysis.

Variable	Men		Women	
	OR (95% CI)	P-value	OR (95% CI)	P-value
Age years	0.98 (0.94, 1.02)	0.3939	0.96 (0.93, 1.00)	**0.0264**
Access to clean water	0.03 (0.00, 0.29)	**0.0019**	0.19 (0.07, 0.52)	**0.0013**
Source of water for drinking	29.75 (3.51, 252.33)	**0.0019**	6.95 (2.37, 20.35)	**0.0004**
Do you use protective measures	3.02 (1.04, 8.79)	**0.0422**	7.46 (2.29, 24.29)	**0.0008**
Any symptoms related to Schistosoma	53.13 (6.08, 464.48)	**0.0003**	18.75 (6.51, 54.04)	**<0.0001**
IL-4 (pg/mL)	1.00 (1.00, 1.00)	0.0590	1.01 (1.01, 1.01)	**<0.0001**
Eosinophil count (x10^9^/L)	10.37 (0.79, 136.56)	0.0754	8.53 (2.30, 31.65)	**0.0014**
Hb (g/dL)	0.96 (0.78, 1.18)	0.7068	0.56 (0.41, 0.77)	**0.0003**
Mcv (fL)	0.99 (0.94, 1.04)	0.6397	0.94 (0.89, 0.99)	**0.0221**
Mch (pg)	0.86 (0.72, 1.02)	0.0835	0.80 (0.70, 0.91)	**0.0010**
MCHC (g/dL)	0.78 (0.55, 1.10)	0.1618	0.72 (0.55, 0.94)	**0.0143**
Lymphocyte count (x10^9^/L)	2.11 (1.04, 4.26)	**0.0378**	2.27 (1.48, 3.48)	**0.0002**
Monocyte count (x10^9^/L)	2.68 (0.51, 14.00)	0.2436	5.38 (1.80, 16.12)	**0.0026**
PLR (x10^9^/L)	1.00 (0.99, 1.01)	0.6221	0.99 (0.98, 1.00)	**0.0063**

**Note:** Statistically significant p-values (<0.05) are shown in bold. OR = Odds Ratio; CI = Confidence Interval.

For Females: Similar to males, access to clean water was protective (OR=0.19, 95% CI: 0.07-0.52, p = 0.0013) and any symptoms were a strong risk factor (OR=18.75, 95% CI: 6.51-54.04, p < 0.0001). Numerous other variables were significant for females that were not for males. These included older age (OR=0.96, p = 0.0264), lower red blood cell indices (hb, mcv, mch, mchc), higher IL-4 (OR=1.01, p < 0.0001), higher eosinophil count (OR=8.53, p = 0.0014), and higher monocyte count (OR=5.38, p = 0.0026). A lower Platelet-to-Lymphocyte Ratio (PLR) was also associated with infection (OR=0.99, p = 0.0063) (Table 3) (Fig 1).

**Fig 1:**
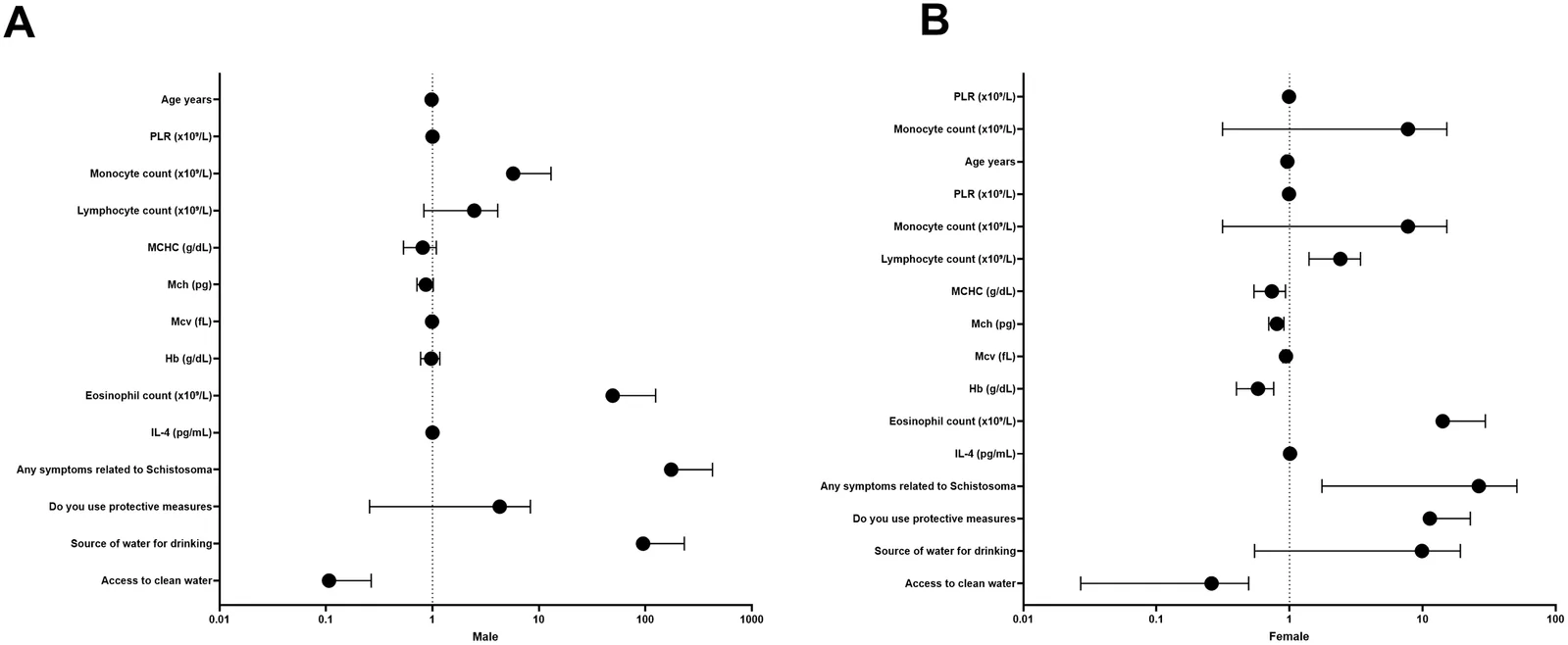
Forest plot for schistosomiasis-related factors associated with male and female sex. **(A)** Forest plot showing the estimated associations of demographic, hematological, immunological, and schistosomiasis-related factors with male sex in univariate analysis. The black circles represent the point estimates, and horizontal lines indicate the corresponding 95% confidence intervals. The vertical dotted line at 0 represents the null value. Variables such as access to clean water, source of drinking water, use of protective measures, symptoms related to *Schistosoma* and lymphocyte count were significantly associated with *Schistosoma* infection (p < 0.05), while age, platelet-to-lymphocyte ratio (PLR), monocyte count, lymphocyte count, mean corpuscular hemoglobin concentration (MCHC), mean corpuscular hemoglobin (MCH), mean corpuscular volume (MCV), hemoglobin (Hb), eosinophil count and interleukin-4 (IL-4) were not associated with *Schistosoma* infection (p > 0.05). **(B)** Forest plot showing the estimated associations of demographic, hematological, immunological, and schistosomiasis-related factors with female sex in Univariate analysis. The black circles represent the point estimates, and horizontal lines indicate the corresponding 95% confidence intervals. The vertical dotted line at 0 represents the null value. Variables assessed included age, access to clean water, platelet-to-lymphocyte ratio (PLR), monocyte count, lymphocyte count, mean corpuscular hemoglobin concentration (MCHC), mean corpuscular hemoglobin (MCH), mean corpuscular volume (MCV), hemoglobin (Hb), eosinophil count, interleukin-4 (IL-4), schistosomiasis-related symptoms, use of protective measures, and source of drinking water (all p < 0.05). Estimates to the right of the null line indicate a positive association with female sex, whereas estimates to the left indicate a negative association. Fig 1(A) and (B) were log_10_-transformed to improve data visualization by reducing skewness and enhancing the comparability of values across a wide range.

### Association in multivariable logistic regression

Table 4 presents the results of the multivariate analysis, which identifies the independent correlates of schistosomiasis after adjusting for other variables in the model.

**Table 4:** Sex differences in correlates of schistosomiasis in multivariate analysis.

Variable	aOR (95% CI)	P-value
**Males**		
Age years	1.01 (0.95, 1.06)	0.8590
Access to clean water	0.03 (0.00, 0.34)	**0.0040**
IL-4 (pg/mL)	1.00 (0.99, 1.00)	0.1360
Hemoglobin (g/dl)	0.96 (0.74, 1.25)	0.7800
**Females**		
Age years	0.99 (0.93, 1.06)	0.8530
Access to clean water	0.11 (0.01, 0.93)	**0.0440**
Any symptoms related to Schistosoma	19.2 (3.17, 116.4)	**0.0010**
IL-4 (pg/mL)	1.01 (1.00, 1.02)	**<0.0001**
Hemoglobin (g/dl)	0.48 (0.28, 0.83)	**0.0080**

**Note:** Statistically significant p-values (<0.05) are shown in bold. OR = Odds Ratio; CI = Confidence Interval.

In males, access to clean water was the only variable independently associated with infection (aOR= 0.03, 95% CI: 0.00–0.34, p = 0.0040), revealing a protective association. Other variables, including age, IL-4 levels, and hemoglobin, were not significantly associated with infection in the adjusted model (Table 4).

In females, several variables remained independently associated with infection. Access to clean water was inversely associated with infection (aOR = 0.11, 95% CI: 0.01–0.93, p = 0.0440). Reporting any symptoms related to schistosomiasis was strongly associated with infection (aOR = 19.2, 95% CI: 3.17–116.4, p = 0.0010). Higher IL-4 levels were also independently associated with infection (aOR = 1.01, 95% CI: 1.00–1.02, p < 0.0001), although the effect size per unit increase was modest. In contrast, hemoglobin levels were inversely associated with infection (aOR = 0.48, 95% CI: 0.28–0.83, p = 0.0080), indicating that lower hemoglobin levels were associated with higher odds of infection. Age was not significantly associated with infection in the adjusted model (Table 4).

## Discussion

The results of this study paint a compelling picture of distinct disease experiences between males and females in this endemic area. The univariate analysis suggests that while both sexes are vulnerable to infection through water exposure, the physiological consequences differ markedly. The female cohort exhibited a stronger and more diverse immune and hematological response, characterized by a Th2-polarized immune signature (elevated IL-4) and significant anemia. This pattern suggests that females may mount a more vigorous, though ultimately ineffective, immune reaction to the parasite, leading to greater hematological compromise.

This finding aligns with the established paradigm of sex-based immunology, where females often mount more robust innate and adaptive immune responses to a variety of pathogens, including helminths [18]. The enhanced Th2 profile observed in women, characterized by elevated IL-4, is consistent with studies showing that estradiol can potentiate Th2-type responses [17]. However, while IL-4 remained independently associated with infection in the multivariable model, the magnitude of effect per unit increase was modest, suggesting that IL-4 should be interpreted as part of a broader immunological response rather than a sole driver of infection risk. Furthermore, the observed anemia in females is consistent with the well-established relationship between chronic schistosomiasis and anemia of inflammation, mediated by iron sequestration and cytokine-driven suppression of erythropoiesis [32,33].

The multivariate analysis further refines these observations. For males, the model identified access to clean water as the only independent correlate of infection, reinforcing the central role of environmental exposure in male infection risk. This finding is consistent with epidemiological studies linking male infection patterns to occupational and behavioral water contact [25]. In contrast, the female, revealed a more complex profile, incorporating both environmental and biological factors. Access to clean water remained inversely associated with infection, while higher IL-4 levels and lower hemoglobin levels were independently associated with infection. Importantly, the inverse association between hemoglobin and infection likely reflects anemia as a consequence of chronic infection rather than a protective factor.

The strong association between reported symptoms and infection observed in females should be interpreted with caution. Given the cross-sectional design and the inclusion of clinical features such as hematuria, which are closely linked to active *S. haematobium* infection, this variable likely reflects disease manifestation rather than an independent risk factor. The large odds ratio observed for this variable may therefore be influenced by the close clinical relationship between symptoms and confirmed infection status.

These findings should be interpreted within the context of the study design and sample size. The multivariable models were restricted to a limited number of predictors based on events-per-variable considerations to reduce overfitting and improve stability. While this approach improved the reliability of the estimates, the relatively small number of outcome events, particularly in sex-stratified analyses may still limit the precision of some estimates.

Taken together, this study supports the concept that urogenital schistosomiasis exhibits sex-associated differences in its correlates. While environmental exposure appears to play a dominant role in males, females reveals a combined profile involving environmental, immunological, and hematological factors. These findings highlight the importance of considering sex as a biological variable in schistosomiasis research and support the need for further longitudinal and mechanistic studies to better understand these differences.

### Strengthens and limitations

A major strength of this study is its sex-stratified analytical approach, which allowed us to identify fundamentally different correlates of *S. haematobium* infection between males and females, a distinction that is often obscured in pooled analyses. The inclusion of both immunological (cytokine) and hematological parameters enabled a comprehensive assessment of the pathophysiological consequences of infection beyond simple parasitological diagnosis. Furthermore, the community-based design enhances the generalizability of our findings to real-world endemic settings, where health system contact may be limited.

This study had a higher enrollment of females (n = 92) compared to males (n = 51), reflecting demographic and logistical realities in the rural setting. While this allowed for detailed exploration of female-specific immunohaematological correlates, it limited the statistical power to detect analogous factors in males. In addition, the cross-sectional design precludes causal inference, and the observed associations between infection and immunohematological parameters cannot establish temporal relationships or distinguish between cause and consequence.

Given the relatively small number of infection events in the sex-stratified analyses, multivariable models were restricted to a limited number of predictors based on events-per-variable considerations to reduce overfitting and improve stability. Although this approach enhanced the reliability of the estimates compared to initial models, some degree of imprecision may persist, particularly for variables closely related to clinical presentation.

Additionally, key exposure variables, including water contact behaviours and use of protective measures, were assessed through self-report. This introduces the possibility of recall bias and social desirability bias, which may have led to misclassification or underestimation of high-risk behaviors, thereby affecting the accuracy of exposure estimates. Furthermore, the classification of water sources as “contaminated” was based on participant-reported contact with natural freshwater bodies and local perceptions of risk, rather than direct environmental or parasitological assessment, which may introduce additional subjectivity.

Finally, while efforts were made to minimize collinearity by excluding closely related variables from the same model, residual confounding cannot be excluded. Future studies incorporating larger sample sizes, longitudinal follow-up, and more detailed exposure assessment including environmental measurements would strengthen causal inference and further clarify the observed sex-associated patterns.

## Conclusion

In conclusion, this study suggests that urogenital schistosomiasis may exhibit sex-associated differences in its correlates within this rural Zambian setting. In males, infection appears to be primarily associated with environmental exposure, particularly access to clean water. In contrast, the female profile reflects a combination of environmental, immunological, and hematological factors, including elevated IL-4 and lower hemoglobin levels. These findings move beyond a uniform understanding of schistosomiasis and support the importance of considering sex as a biological variable in both research and control strategies. However, given the cross-sectional design, relatively small sample size, and limitations inherent to sex-stratified analyses, these findings should be interpreted with caution. Future studies incorporating larger sample sizes, longitudinal follow-up, and more comprehensive immunological profiling are needed to confirm these observations and to better understand the mechanisms underlying sex-associated differences in schistosomiasis.

**Key points**

- **Distinct Sex-Specific Profiles:** The study identifies fundamentally different correlates of *S. haematobium* infection between sexes. Male infection is primarily linked to environmental exposure (lack of clean water), while female infection is characterized by a pronounced immunological (elevated IL-4) and hematological (anemia) response.
- **Female-Specific Immune-Hematological Link:** In females, infection is independently associated with a stronger Th2-polarized immune response (higher IL-4) and significant anemia (lower hemoglobin), suggesting a more vigorous immunopathological reaction that contributes to key morbidity.
- **Implications for Differential Management:** The findings underscore the necessity for sex-differentiated approaches in both clinical assessment, where women may require closer monitoring for anemia, and in public health control strategies that address distinct risk profiles.

## Supporting information

S1 STROBE Checklist STROBE Statement—Checklist of items that should be included in reports of cross-sectional studies. (DOCX)

S1 Data Schistosomiasis data. (XLSX)
