# Supplementary material for: Sex-specific correlates of Schistosoma haematobium infection in a rural Zambian setting: A pilot study focusing on immune and hematological factors
Source: PLOS Glob Public Health. 2026 Nov 4;6(11):e0005692. doi: 10.1371/journal.pgph.0005692 (PMC13734442; doi:10.1371/journal.pgph.0005692)
Supplement: Supplementary file 2 [file pgph.0005692.s003.docx]

February 5, 2026

Dear Editor,

**RE: REVIEW REPORT FOR MANUSCRIPT PGPH-D-25-03800**

Thank you for giving me the opportunity to review the manuscript titled **“Sex-Specific Correlates of Schistosoma haematobium Infection in a Rural Zambian Setting: A pilot study Focusing on Immune and Hematological Factors”.** This article provides interesting information on association between sex diversity and immune, hematological, and environmental factors associated with *Schistosoma haematobium* infection. I comment the authors for this study. However, I have several concerns for the authors to consider.

1. In lines 100-101, the authors need to give a brief description of the method used to identify ova in urine. Asa general standard practice, since the authors are not the ones doing initial description of the diagnostic technique, they should include a reference of the author/s who did the initial description and publication of the method.
2. In lines 102-104 indicate the Elisa kit that was used to determine plasma concentration of cytokines and the manufacturer. This is important for reproducibility of other scientists who might want to do similar work in future after you publish your findings.
3. The authors are too brief in the introduction section; they should provide more literature review of on previous related studies concerning immune response profile even against other *Schistosoma spp* that infect humans.
4. For the study participants, what is the justification of having a significantly high number of females than males? Could an equal number of males/females have provided a different view of the results.
5. On Table 1:
a) How was data on “Signs and symptoms” collected among study participants, which among the several signs/symptoms of urogenital schistosomiasis were considered, was this evaluation of the signs/symptoms done by a physician! Need for clarification.
b) The word “Schistosomia” what does it mean? I guess the authors meant “schistosomiasis” rectify the spelling error.
c) The percentage of schistosomiasis cases among males is wrong 18 out of 51 should be 35.3%!!!and for females 38/92 = 38%, same error applies to negative cases. The calculation in this table needs a close look and review.
d) On the parameter of “protective measures”, which consideration was given among the many measures that protect against infection like wearing boots, hand gloves, boiling bathing water etc, kindly specific. Same case applies to engage in activities with contaminated water, how are members of the community able to determine that the water they come into contact with is contaminated with cercariae (the larvae stage that infects definitive hosts)!!

In general, there is need to provide a clear description on methods od data collection otherwise as it is the data that is being presented here there is minimal information on how it was obtained.
