# Supplementary material for: Sex-specific correlates of Schistosoma haematobium infection in a rural Zambian setting: A pilot study focusing on immune and hematological factors
Source: PLOS Glob Public Health. 2026 Nov 4;6(11):e0005692. doi: 10.1371/journal.pgph.0005692 (PMC13734442; doi:10.1371/journal.pgph.0005692)
Supplement: Supplementary file 3 [file pgph.0005692.s004.docx]

**Point by Point responses to reviewers’ comments**

February 5, 2026

Dear Editor,

**RE: REVIEW REPORT FOR MANUSCRIPT PGPH-D-25-03800**

Thank you for giving me the opportunity to review the manuscript titled **“Sex-Specific Correlates of Schistosoma haematobium Infection in a Rural Zambian Setting: A pilot study Focusing on Immune and Hematological Factors”.** This article provides interesting information on association between sex diversity and immune, hematological, and environmental factors associated with *Schistosoma haematobium* infection. I comment the authors for this study. However, I have several concerns for the authors to consider.

We thank the editors and reviewers for their time and insightful feedback, which has significantly improved the rigor and quality of our manuscript. We have revised the paper to address all the comments raised. Our point-by-point responses are provided in blue below, and the corresponding changes have been incorporated into the manuscript. Both a clean copy and a version with tracked changes have been uploaded.

1. In lines 100-101, the authors need to give a brief description of the method used to identify ova in urine. Asa general standard practice, since the authors are not the ones doing initial description of the diagnostic technique, they should include a reference of the author/s who did the initial description and publication of the method.

**Response**

We thank the reviewer for this insightful suggestion. We have revised**. “A 10 mL of well-mixed urine was centrifuged at 1500–2000 rpm for 5 minutes. The supernatant was discarded, and the entire sediment was transferred to a glass slide and examined systematically under light microscopy at X10 and X40 magnification for the presence of characteristic terminal-spined S. haematobium eggs. All examinations were performed independently by two trained laboratory technologists; in cases of disagreement, a third technologist served as the tie breaker.”**

2. In lines 102-104 indicate the Elisa kit that was used to determine plasma concentration of cytokines and the manufacturer. This is important for reproducibility of other scientists who might want to do similar work in future after you publish your findings.

**Response**

We thank the reviewer for this insightful suggestion. We have revised the Methods section to include the full details of the ELISA kit used, including the kit name, manufacturer, and country of origin. Plasma concentrations of the cytokines IL-4 and IL-10 were determined using commercially available, validated enzyme-linked immunosorbent assay (ELISA) kits **Elabscience (Elabscience Biotechnology Inc., China), in accordance with the manufacturer’s instructions.**

3. The authors are too brief in the introduction section; they should provide more literature review of on previous related studies concerning immune response profile even against other *Schistosoma spp* that infect humans.

**Response**

We thank the reviewer for this insightful suggestion. We have added as follows **“Studies across Schistosoma species have revealed both conserved and species-specific immune features. Research on S. mansoni has established the fundamental paradigm that egg deposition drives a robust Th2-polarized response, with IL-4 playing a central role in granuloma formation and fibrosis [8]. Murine models of S. mansoni infection have demonstrated that signal transducer and activator of transcription 6 (STAT6) and IL-4 receptor alpha signaling are essential for granuloma development, as their ablation markedly reduces pathology [9]. More recently, investigations into S. japonicum have shown that glucocorticoid receptor activation can suppress Th2 responses by downregulating the transcription factors Gata3 and Pparg, offering potential therapeutic insights applicable across schistosome species [10].**

**The cytokine milieu in schistosomiasis extends beyond the classic Th2 axis. Tumor necrosis factor (TNF) has been implicated in both limiting liver pathology and promoting adult parasite survival in S. mansoni infections, suggesting a complex host-parasite co-evolution where inflammatory mediators serve dual roles [11]. Studies of circulating cytokines in S. haematobium-endemic populations in Zimbabwe have revealed that egg-positive individuals exhibit significantly higher levels of IL-2, IFN-γ, and IL-23, while egg-negative individuals demonstrate elevated IL-10, IL-4, IL-13, and IL-21 [12]. This mixed Th1/Th2/regulatory profile in patent infection, contrasting with a Th2-dominated suite in egg-negative individuals, underscores the dynamic nature of the host response and the importance of immunoregulation in determining infection outcomes.**

**Moreover, hematological alterations are also a recognized feature of chronic schistosomiasis. Anemia in S. mansoni and S. haematobium has been attributed to chronic inflammation, iron sequestration mediated by hepcidin, occult blood loss, and bone marrow suppression driven by inflammatory cytokines [13,14]. However, most immunopathological studies of human schistosomiasis focus on single-sex cohorts or fail to stratify by biological sex, despite mounting evidence that sex hormones modulate cytokine production, antibody responses, and eosinophil activity.”**

4. For the study participants, what is the justification of having a significantly high number of females than males? Could an equal number of males/females have provided a different view of the results.

**Response**

We thank the reviewer for raising this important methodological point regarding the sex distribution of our study participants.

We have revised the methods section “**This sex distribution reflects the demographic and practical realities of the study setting, where women were more frequently available at home during daytime recruitment visits. Enrollment was voluntary and no potential participants were excluded based on sex; the imbalance arose naturally from community availability and willingness to participate. While this provided adequate power to explore female-specific correlates, a priority given the focus on immunohaematological manifestations like anemia, we acknowledge that the smaller male subgroup may limit the detection of more subtle correlates in men**”.

We have also noted this as a limitation. “**This study had a higher enrollment of females (n=92) compared to males (n=51), reflecting demographic and logistical realities in the rural setting. While this allowed for detailed exploration of female-specific immunohaematological correlates, it limited the statistical power to detect analogous factors in males. A balanced, sex-matched design in future studies would enhance comparability and enable more robust detection of sex-specific pathophysiological pathways**.”

5. On Table 1:
a) How was data on “Signs and symptoms” collected among study participants, which among the several signs/symptoms of urogenital schistosomiasis were considered, was this evaluation of the signs/symptoms done by a physician! Need for clarification.

**Response**

We thank the reviewer for the observation. We have revised and added in line 102-131. “**Assessment of Signs and Symptoms: Clinical signs and symptoms of urogenital schistosomiasis were assessed through age-appropriate, standardized approaches conducted by trained study clinicians (clinical officers and medical doctors). Given that the study population included both children and adults (age range 5-55 years, median 15 years), special consideration was given to adolescent participants to ensure comfort and appropriate communication during assessments.**

**Symptom Assessment: All participants or their caregivers (for younger children) underwent a structured interview administered by trained research nurses in the local language (Tonga/Lozi). The interview inquired about the presence of classic urogenital schistosomiasis symptoms in the preceding four weeks, including: visible hematuria (blood in urine), dysuria (painful urination), frequent urination, and suprapubic discomfort or report of these symptoms by a study participant. For adolescent female participants (≥12 years), age-appropriate questions were carefully framed to inquire about potential symptoms of female genital schistosomiasis, including vaginal discharge, genital itching, and contact bleeding, with a parent or guardian present if the participant and guardian consented. For younger children (<12 years), symptom history was obtained primarily from the parent or guardian.**

**Clinical Examination: Each participant received a brief physical examination by a study clinician in the presence of a same-gender chaperone. For all participants, this included assessment for visible hematuria and suprapubic tenderness on palpation. For adolescent participants, examinations were conducted with strict attention to privacy and comfort.**

**Definition of Any Signs/Symptoms: For the purpose of analysis, participants were classified as having 'any signs/symptoms' if they reported at least one symptom during the age-appropriate interview OR had at least one clinical sign identified on examination that was attributable to urogenital schistosomiasis. This composite variable captured both symptomatic presentations (e.g., dysuria reported by an adolescent) and objective findings (e.g., visible hematuria observed by the clinician).**

**All clinical assessments were conducted by trained study clinicians, and standardized training was provided to ensure consistency in symptom inquiry and examination techniques across the study team, with additional training on age-appropriate communication with adolescent participants**.”

b) The word “Schistosomia” what does it mean? I guess the authors meant “schistosomiasis” rectify the spelling error.

**Response**

We thank the reviewer for the observation. We have reviewed and revised the spelling errors.

c) The percentage of schistosomiasis cases among males is wrong 18 out of 51 should be 35.3%!!!and for females 38/92 = 38%, same error applies to negative cases. The calculation in this table needs a close look and review.

**Response**

Thank you for your careful review of our manuscript and for identifying the specific errors in Table 1. You are absolutely correct, and we appreciate you bringing this to our attention.

We have now completely redone the table. We have carefully recalculated all percentages and cross-checked every figure against the raw data. The errors you pointed out, specifically the miscalculation of the male schistosomiasis cases (18/51 = 35.3%, not the previously reported figure) and the corresponding female percentage (38/92 = 38%) have been corrected. We have also applied the same rigorous check to all other cells, to ensure the entire table is now accurate and consistent.

Additionally, we have reviewed all tables and added appropriate units to every variable:

Table 1: Added units for all continuous variables (age, IL-4, IL-10, eosinophil count, RBC, Hb, MCV, MCH, MCHC, neutrophil count, lymphocyte count, monocyte count, platelet count)

Table 2: Added units to all variable names (e.g., "IL-4 (pg/mL)", "Hemoglobin (g/dL)", "Eosinophil count (×10⁹/L)")

Table 3: Standardized units to match Table 2, corrected IL-4 unit to pg/mL for consistency

d) On the parameter of “protective measures”, which consideration was given among the many measures that protect against infection like wearing boots, hand gloves, boiling bathing water etc, kindly specific. Same case applies to engage in activities with contaminated water, how are members of the community able to determine that the water they come into contact with is contaminated with cercariae (the larvae stage that infects definitive hosts)!!

Response

Thank you for this thoughtful and important query. You have raised two critical points regarding the operational definitions of key exposure variables that require clarification in our manuscript. Regarding "protective measures": You are absolutely correct that this term is too vague in its current form. We have revised and have added.

**“Protective Measures:**

**Participants were asked about their use of personal protective behaviors when engaging in activities that involved contact with surface water (rivers, streams, dams). Based on preliminary community engagement and known local practices, the following specific measures were inquired about:**

1. **Footwear use: Wearing closed shoes, boots, or sandals when entering water**
2. **Glove use: Wearing protective gloves when washing clothes or dishes in river water**
3. **Water handling practices: Allowing river water to settle before use, or using containers to collect water without full body immersion**
4. **Avoidance behaviors: Deliberately avoiding water contact during peak cercarial shedding times (mid-day to early afternoon) when community knowledge indicates higher risk**

**Participants were asked to report their typical practices and were categorized based on their response to a single question: "How often do you use any protective measures (such as those we discussed) when you come into contact with river or stream water?" Response options were: "Always" (consistently use one or more measures), "Sometimes" (occasionally use measures), or "Never" (do not use any protective measures). This composite variable was used in the analysis as "protective measures."**

In general, there is need to provide a clear description on methods od data collection otherwise as it is the data that is being presented here there is minimal information on how it was obtained.

Response

Thank you for this thoughtful and important query. We have carefully addressed all comments pertaining data collection refer to manuscript line 102-131.
