# Supplementary material for: Sex-specific correlates of Schistosoma haematobium infection in a rural Zambian setting: A pilot study focusing on immune and hematological factors
Source: PLOS Glob Public Health. 2026 Nov 4;6(11):e0005692. doi: 10.1371/journal.pgph.0005692 (PMC13734442; doi:10.1371/journal.pgph.0005692)
Supplement: Supplementary file 4 [file pgph.0005692.s005.docx]

**Response to reviewers’ comments**

We thank the Editor and the reviewers for their careful evaluation of our manuscript and for their constructive and insightful comments. We have thoroughly revised the manuscript in response to all points raised and have addressed each comment in detail below. We believe that these revisions have significantly strengthened the clarity, rigor, and overall quality of the manuscript.

Reviewer #2: The present study aims to identify and compare the sex-specific immune, hematological, and environmental factors associated with Schistosoma haematobium infection in a rural Zambian setting. This study shows that urogenital schistosomiasis does not affect males and females in the same way. In males the disease is largely driven by external exposure-related factors. In females it presents with a distinct immunopathological profile, marked by a strong Th2 immune response and notable anemia. The study findings provide valuable insights in treating schistosomiasis in men and women. However, there are still issues in the manuscript that need to be resolved before it can be considered ready for publication as follows:

[A] The multivariate analysis (Table 3) shows implausibly wide confidence intervals for some variables, suggesting model instability, likely due to small sample size, collinearity, or overfitting. My recommendations:

- (i) Acknowledge in the results that these wide intervals indicate instability and interpret findings cautiously. (ii) Reassess the number of variables included relative to the sample size (e.g., limiting variables based on events-per-variable guidelines). (iii) Report the number of outcome events per variable to support the model’s validity.

**Response**

We thank the reviewer for this important and insightful comment. In response, we have substantially revised the multivariable analysis to improve model stability and interpretability. We agree that the presence of very wide confidence intervals for several variables in the multivariate analysis (Table 3) indicates potential model instability. In response to this concern, we have made the following revisions:

- We have explicitly stated in the Results and Discussion sections that the wide confidence intervals reflect statistical instability and that these estimates should be interpreted cautiously.

- We reviewed the number of variables included in the multivariate models in relation to the number of outcome events. Following established recommendations (e.g., ≥10 events per variable). First, we reduced the number of predictors included in the multivariable models based on events-per-variable (EPV) considerations. In both males and female subgroup, which included 38 vs 16 infection events, the number of predictors was restricted to a parsimonious set selected on the basis of biological plausibility, strength of univariate association, and avoidance of collinearity. Second, closely related variables (e.g., multiple red cell indices and overlapping exposure variables) were not included simultaneously in the same model to minimize collinearity. Third, the revised models show improved stability, with substantially narrower confidence intervals compared to the initial analysis. However, we acknowledge that some estimates, particularly for the “symptoms” variable, remain relatively large, reflecting the close relationship between clinical presentation and infection status in cross-sectional data.

We have revised the Methods, Results and Discussion sections to explicitly describe the model selection approach and to interpret the findings cautiously in light of these considerations.

[B] Methodological clarification: While “protective measures” are well described, the variable “Engage in activities with contaminated water” remains unclear. It is uncertain how participants identified water as contaminated. My recommendation:

- The authors should clarify how this variable was defined and measured. If based on participant perception, this should be explicitly stated as a limitation.

**Response**

We thank the reviewer for this important observation. We acknowledge that the term “contaminated water” may be ambiguous if not clearly defined. In our study, this variable referred to participant-reported frequency of contact with natural freshwater bodies (e.g., rivers, streams, and dams), which are recognized endemic transmission sites for Schistosoma haematobium in the study area. We have revised the Methods section to clarify this definition. We also acknowledge that this measure was based on participant self-report and local understanding of risky water sources, which may introduce subjectivity. This limitation has now been explicitly stated in the Strengths and Limitations section.

[C] Methodological clarification: Details about the ELISA kits are mentioned but not consistently integrated into the manuscript. My recommendation:

- Ensure that the manufacturer’s details are clearly and consistently reported in the Methods section, including catalog numbers where available.

**Response**

We thank the reviewer for highlighting this issue. We agree that consistent and detailed reporting of assay reagents is important for reproducibility. We have revised the Methods section to ensure that the ELISA kit manufacturer details are clearly and consistently presented, and we have included catalog numbers where available.

[D] Strengths and limitations: The strengths and limitations section is well developed but does not address potential bias in self-reported data. My recommendation:

- The manuscript would benefit from a brief statement recognizing that reliance on self-reported behaviors introduces the potential for recall and social desirability bias, potentially affecting the validity of exposure estimates.

**Response**

We thank the reviewer for this insightful comment. We agree that reliance on self-reported behavioral data may introduce recall and social desirability bias, which could affect the accuracy of exposure estimates. We have revised the Strengths and Limitations section to explicitly acknowledge this potential source of bias and its possible impact on the interpretation of exposure-related findings.

Overall, I approve publication once the above-mentioned issues raised is adequately addressed.
