# Supplementary material for: Sex-specific correlates of Schistosoma haematobium infection in a rural Zambian setting: A pilot study focusing on immune and hematological factors
Source: PLOS Glob Public Health. 2026 Nov 4;6(11):e0005692. doi: 10.1371/journal.pgph.0005692 (PMC13734442; doi:10.1371/journal.pgph.0005692)
Supplement: Supplementary file 5 [file pgph.0005692.s006.docx]

Reviewer #2: The authors have adequately addressed all my comments and issues raised.

Reviewer #3: General comment
In the manuscript, Sex-Specific Correlates of Schistosoma haematobium Infection in a Rural Zambian Setting: A Pilot Study Focusing on Immune and Hematological Factors,” Masenga et al. aim to identify and compare sex-specific immune, hematological, and environmental correlates of Schistosoma haematobium infection in a rural Zambian population. The topic is interesting and potentially valuable, particularly given the importance of sex-specific analyses in schistosomiasis research. However, I have several significant concerns regarding the statistical analysis and study design. My primary concern relates to the validity of the baseline comparisons, which substantially affects confidence in the downstream analyses. At present, I believe the manuscript requires major revision, and if these concerns cannot be adequately addressed, publication may not be appropriate.

Concerns

1 Potential inconsistency in p-values reported in Table 1: In Table 1, I assume the authors used a statistical test for comparing categorical variables between male and female groups (possibly a two-proportion Z-test). I independently checked several reported p-values and found that some match the manuscript (e.g., Schistosomiasis, p = 0.4806; Have you ever been diagnosed, p = 0.6183), but many others do not. Examples include: \\
Access to clean water; Yes (my calculation: p = 0.3922) \\
Source of drinking water; Tap (my calculation: p = 0.3236)
Signs/Symptoms; Yes (my calculation: p = 0.4451) \\
Education; Not Schooled (my calculation: p = 0.6822) \\
Protective measures; Always (my calculation: p = 0.2238) \\

These discrepancies raise concerns about how the p-values were computed and reported. The authors should clearly specify which statistical tests were used for each categorical variable and verify all reported p-values in Table 1. Since Table 1 serves as the foundation for later analyses, any errors here may affect the validity of the subsequent results and conclusions. \\

**Response**

We thank the reviewer for this observation. The p-values recommended by the reviewer were those reported in the initial submission of the manuscript. However, following a suggestion raised during the previous review cycle, we revised the reported p-values. We have now corrected the values as suggested by the reviewer and carefully verified their consistency throughout Table 1.

In addition, if the corrected p-values are indeed such high and non-significant for all baseline characteristics, this weakens the motivation for the manuscript's central objective of investigating sex-specific differences. The study is framed around identifying meaningful differences between male and female profiles; however, if the baseline comparisons do not support notable sex-based differences, the rationale for emphasizing sex-specific analyses should be more carefully justified.

**Response**

We thank the reviewer for this important observation. We agree that the rationale for the sex-specific analyses should be more clearly articulated. However, the absence of statistically significant differences in baseline characteristics between males and females does not necessarily preclude the investigation of sex-specific associations. The primary objective of the study was not to demonstrate differences in baseline characteristics (Cho-square) by sex, but rather to investigate whether the relationships between the study variables and the outcome differed according to sex using logistic regression analyses.

We have also ensured that the interpretation of the findings is appropriately cautious and focused on the observed sex-specific associations rather than on baseline comparisons alone.

2. Unclear variable selection for multivariate analysis: It is unclear why only the variables age, access to clean water, use of protective measures, symptoms, and lymphocyte count were selected for the multivariate analysis (at least for the male subgroup). \\
Based on Table 2, multiple variables showed low p-values and significant univariate associations. The manuscript does not clearly explain the rationale for including some variables while excluding others. Were excluded variables omitted because of collinearity, redundancy, missing data, or model instability? If so, this should be explicitly stated. The authors should provide a clear variable selection strategy for the multivariate models.

**Response**

We thank the reviewer for this important comment. Variable selection for the multivariable logistic regression model was based on statistical significance (p<0.05) in the unadjusted logistic regression analysis. To assess sex-specific differences, the same set of selected variables was entered into the adjusted models for males and females. This approach was adopted to minimize overadjustment and model instability while ensuring that comparable variables were used across the sex-specific models. We have revised the methods section statistical analysis section to clarify.

3. Presentation of univariate associations in Table 2: Table 2 presents the associations between schistosomiasis status and each variable using odds ratios. While this is informative, the results would be easier to interpret with complementary visual representations. I recommend including visual summaries such as: side-by-side bar plots for categorical variables,
stacked bar plots for proportions, or
side-by-side boxplots for continuous variables. \\
Such figures would improve readability and help readers better understand the observed differences.

**Response**

We thank the reviewer for the valuable suggestion. We agree that presenting the data visually enhances clarity and interpretability. Accordingly, we have revised the manuscript and have now included forest plots to display the odds ratios and 95% confidence intervals for all variables.

Specifically:

Figure 1 now presents the forest plot for the male subgroup.

Figure 2 now presents the forest plot for the female subgroup.

4. (Minor) Table organization: I recommend separating Table 1 into two tables: one for categorical variables (frequency and percentage), and
one for continuous variables (median and IQR).

**Response**

We thank the reviewer for this helpful suggestion. We have revised the table organization by separating Table 1 into two tables: one presenting categorical variables as frequencies and percentages, and the other presenting continuous variables as medians and interquartile ranges (IQR).

5. (Minor) Lines 204–206: the authors mention the use of Mann–Whitney U tests and Chi-square tests. I recommend explicitly labeling the statistical test used for each analysis in the tables or other appropriate places.

**Response**

We thank the reviewer for this helpful suggestion. We have addressed this by separating the baseline characteristics into two tables. Table 1 presents categorical variables as frequencies and percentages, with the Chi-square test used for comparisons, while Table 2 presents continuous variables as medians and interquartile ranges (IQRs), with the Mann–Whitney U test used for comparisons. The statistical tests have also been clearly indicated in the respective table footnotes.
