# Supplementary material for: Sex-specific correlates of Schistosoma haematobium infection in a rural Zambian setting: A pilot study focusing on immune and hematological factors
Source: PLOS Glob Public Health. 2026 Nov 4;6(11):e0005692. doi: 10.1371/journal.pgph.0005692 (PMC13734442; doi:10.1371/journal.pgph.0005692)
Supplement: Supplementary file 6 [file pgph.0005692.s007.docx]

We sincerely thank the Editor and Reviewers for the time and effort devoted to reviewing our manuscript and for their valuable comments and suggestions. We have carefully considered and addressed all the comments, and the manuscript has been revised accordingly. We believe these revisions have improved the clarity, presentation, and overall quality of the manuscript.

1. Text formatting in Tables 1 and 2: The table headings and p-values appear inconsistently formatted, with some text shown in black or gray and in regular or bold font. Please check whether this is a formatting issue or an intentional distinction. If the formatting is intentional, please clarify its purpose and apply it consistently throughout the tables.

**Response**
Thank you for this observation. The inconsistent formatting of the table headings and p-values was unintentional and resulted from formatting inconsistencies. We have corrected the formatting in Tables 1 and 2 to ensure consistency throughout.

2. Figures 1 and 2: Using the same scale for all measures makes the confidence intervals of many variables invisible. Consider revising the graphical format or scale to improve readability. If feasible, I would also suggest presenting the male and female results side by side in a single figure, which would facilitate direct comparison between the two groups.

**Response**
Thank you for this helpful suggestion. We have revised the figures to improve readability and facilitate comparison between male and female results. The two figures have been combined into a single figure with panels A and B, presenting the male and female results side by side. The figures have also been log₁₀-transformed to reduce skewness and improve the visualisation and comparability of estimates across a wide range of values.
