# Supplementary material for: Sex-specific correlates of Schistosoma haematobium infection in a rural Zambian setting: A pilot study focusing on immune and hematological factors
Source: PLOS Glob Public Health. 2026 Nov 4;6(11):e0005692. doi: 10.1371/journal.pgph.0005692 (PMC13734442; doi:10.1371/journal.pgph.0005692)
Supplement: Peer review history

# Peer review: Decision Letter 0

**DOI:** 10.1371/journal.pgph.0005692.r001

**Authors:** Johanna Pruller

9 Feb 2026

PGPH-D-25-03800

Sex-Specific Correlates of Schistosoma haematobium Infection in a Rural Zambian Setting: A pilot study Focusing on Immune and Hematological Factors

PLOS Global Public Health

Dear Dr. Masenga,

Thank you for submitting your manuscript to PLOS Global Public Health. After careful consideration, we feel that it has merit but does not fully meet PLOS Global Public Health’s publication criteria as it currently stands. Therefore, we invite you to submit a revised version of the manuscript that addresses the points raised during the review process.

==============================

Please note that we have only been able to secure a single reviewer to assess your manuscript. We are issuing a decision on your manuscript at this point to prevent further delays in the evaluation of your manuscript. Please be aware that the editor who handles your revised manuscript might find it necessary to invite additional reviewers to assess this work once the revised manuscript is submitted. However, we will aim to proceed on the basis of this single review if possible.

Could you please revise the manuscript to carefully address the concerns raised?

==============================

Please submit your revised manuscript by Mar 26 2026 11:59PM. If you will need more time than this to complete your revisions, please reply to this message or contact the journal office at globalpubhealth@plos.org. When you're ready to submit your revision, log on to https://www.editorialmanager.com/pgph/ and select the 'Submissions Needing Revision' folder to locate your manuscript file.

Please include the following items when submitting your revised manuscript:

- A letter that responds to each point raised by the editor and reviewer(s). You should upload this letter as a separate file labeled 'Response to Reviewers'.
- A marked-up copy of your manuscript that highlights changes made to the original version. You should upload this as a separate file labeled 'Revised Manuscript with Track Changes'.
- An unmarked version of your revised paper without tracked changes. You should upload this as a separate file labeled 'Manuscript'.

Guidelines for resubmitting your figure files are available below the reviewer comments at the end of this letter.

We look forward to receiving your revised manuscript.

Kind regards,

Johanna Pruller, Ph.D.

Senior Editor

PLOS Global Public Health

Journal Requirements:

1. Please ensure that your Ethics Statement is available in its entirety at the beginning of your Methods section, under a subheading 'Ethics Statement'.

If the reviewer comments include a recommendation to cite specific previously published works, please review and evaluate these publications to determine whether they are relevant and should be cited. There is no requirement to cite these works unless the editor has indicated otherwise.

Additional Editor Comments (if provided):

[Note: HTML markup is below. Please do not edit.]

Reviewers' comments:

Reviewer's Responses to Questions

**Comments to the Author**

1. Does this manuscript meet PLOS Global Public Health’s publication criteria?

Reviewer #1: Partly

**********

2. Has the statistical analysis been performed appropriately and rigorously?

Reviewer #1: No

**********

3. Have the authors made all data underlying the findings in their manuscript fully available (please refer to the Data Availability Statement at the start of the manuscript PDF file)?

The PLOS Data policy

Reviewer #1: Yes

**********

4. Is the manuscript presented in an intelligible fashion and written in standard English?

Reviewer #1: Yes

**********

Reviewer #1: This article provides interesting information on association between sex diversity and immune, hematological, and environmental factors associated with Schistosoma haematobium infection. I comment the authors for this study. However, I have several concerns for the authors to consider, see attachement

**********

what does this mean?). If published, this will include your full peer review and any attached files.

**Do you want your identity to be public for this peer review?**  If you choose “no”, your identity will remain anonymous but your review may still be made public.

For information about this choice, including consent withdrawal, please see our Privacy Policy

Reviewer #1: No

**********

[NOTE: If reviewer comments were submitted as an attachment file, they will be attached to this email and accessible via the submission site. Please log into your account, locate the manuscript record, and check for the action link "View Attachments". If this link does not appear, there are no attachment files.]

Attachment Submitted filename: REVIEW REPORT FOR MANUSCRIPT PGPH-D-25-03800 Feb 5, 2026.docx

# Author comment: Author response to Decision Letter 1

**DOI:** 10.1371/journal.pgph.0005692.r002

23 Feb 2026

Attachment Submitted filename: Response to Reviewers (1).docx

# Peer review: Decision Letter 1

**DOI:** 10.1371/journal.pgph.0005692.r003

**Authors:** Xin Sun

8 Apr 2026

PGPH-D-25-03800R1

Sex-Specific Correlates of Schistosoma haematobium Infection in a Rural Zambian Setting: A pilot study Focusing on Immune and Hematological Factors

PLOS Global Public Health

Dear Dr. Masenga,

Thank you for submitting your manuscript to PLOS Global Public Health. After careful consideration, we feel that it has merit but does not fully meet PLOS Global Public Health’s publication criteria as it currently stands. Therefore, we invite you to submit a revised version of the manuscript that addresses the points raised during the review process.

Please submit your revised manuscript by May 23 2026 11:59PM. If you will need more time than this to complete your revisions, please reply to this message or contact the journal office at globalpubhealth@plos.org. When you're ready to submit your revision, log on to https://www.editorialmanager.com/pgph/ and select the 'Submissions Needing Revision' folder to locate your manuscript file.

Please include the following items when submitting your revised manuscript:

- A letter that responds to each point raised by the editor and reviewer(s). You should upload this letter as a separate file labeled 'Response to Reviewers'.
- A marked-up copy of your manuscript that highlights changes made to the original version. You should upload this as a separate file labeled 'Revised Manuscript with Track Changes'.
- An unmarked version of your revised paper without tracked changes. You should upload this as a separate file labeled 'Manuscript'.

Guidelines for resubmitting your figure files are available below the reviewer comments at the end of this letter.

As the corresponding author, your ORCID iD is verified in the submission system and will appear in the published article. PLOS supports the use of ORCID, and we encourage all coauthors to register for an ORCID iD and use it as well. Please encourage your coauthors to verify their ORCID iD within the submission system before final acceptance, as unverified ORCID iDs will not appear in the published article. *Only*  the individual author can complete the verification step; PLOS staff *cannot*  verify ORCID iDs on behalf of authors.

We look forward to receiving your revised manuscript.

Kind regards,

Xin Sun, PhD

Staff Editor

PLOS Global Public Health

Journal Requirements:

If the reviewer comments include a recommendation to cite specific previously published works, please review and evaluate these publications to determine whether they are relevant and should be cited. There is no requirement to cite these works unless the editor has indicated otherwise.

Additional Editor Comments (if provided):

[Note: HTML markup is below. Please do not edit.]

Reviewers' comments:

Reviewer's Responses to Questions

**Comments to the Author**

Reviewer #1: All comments have been addressed

Reviewer #2: All comments have been addressed

**********

publication criteria?

Reviewer #1: Yes

Reviewer #2: Yes

**********

3. Has the statistical analysis been performed appropriately and rigorously?

Reviewer #1: Yes

Reviewer #2: Yes

**********

4. Have the authors made all data underlying the findings in their manuscript fully available (please refer to the Data Availability Statement at the start of the manuscript PDF file)?

The PLOS Data policy

Reviewer #1: Yes

Reviewer #2: Yes

**********

5. Is the manuscript presented in an intelligible fashion and written in standard English?

Reviewer #1: Yes

Reviewer #2: Yes

**********

Reviewer #1: No further comments

Reviewer #2: The present study aims to identify and compare the sex-specific immune, hematological, and environmental factors associated with Schistosoma haematobium infection in a rural Zambian setting. This study shows that urogenital schistosomiasis does not affect males and females in the same way. In males the disease is largely driven by external exposure-related factors. In females it presents with a distinct immunopathological profile, marked by a strong Th2 immune response and notable anemia. The study findings provide valuable insights in treating schistosomiasis in men and women. However, there are still issues in the manuscript that need to be resolved before it can be considered ready for publication as follows:

[A] The multivariate analysis (Table 3) shows implausibly wide confidence intervals for some variables, suggesting model instability, likely due to small sample size, collinearity, or overfitting. My recommendations:

- (i) Acknowledge in the results that these wide intervals indicate instability and interpret findings cautiously. (ii) Reassess the number of variables included relative to the sample size (e.g., limiting variables based on events-per-variable guidelines). (iii) Report the number of outcome events per variable to support the model’s validity.

[B] Methodological clarification: While “protective measures” are well described, the variable “Engage in activities with contaminated water” remains unclear. It is uncertain how participants identified water as contaminated. My recommendation:

- The authors should clarify how this variable was defined and measured. If based on participant perception, this should be explicitly stated as a limitation.

[C] Methodological clarification: Details about the ELISA kits are mentioned but not consistently integrated into the manuscript. My recommendation:

- Ensure that the manufacturer’s details are clearly and consistently reported in the Methods section, including catalog numbers where available.

[D] Strengths and limitations: The strengths and limitations section is well developed but does not address potential bias in self-reported data. My recommendation:

- The manuscript would benefit from a brief statement recognizing that reliance on self-reported behaviors introduces the potential for recall and social desirability bias, potentially affecting the validity of exposure estimates.

Overall, I approve publication once the above-mentioned issues raised is adequately addressed.

**********

what does this mean?). If published, this will include your full peer review and any attached files.

**Do you want your identity to be public for this peer review?**  If you choose “no”, your identity will remain anonymous but your review may still be made public.

For information about this choice, including consent withdrawal, please see our Privacy Policy

Reviewer #1: No

Reviewer #2: No

**********

[NOTE: If reviewer comments were submitted as an attachment file, they will be attached to this email and accessible via the submission site. Please log into your account, locate the manuscript record, and check for the action link "View Attachments". If this link does not appear, there are no attachment files.]

# Author comment: Author response to Decision Letter 2

**DOI:** 10.1371/journal.pgph.0005692.r004

29 Apr 2026

Attachment Submitted filename: Response to Reviewers (4).docx

# Peer review: Decision Letter 2

**DOI:** 10.1371/journal.pgph.0005692.r005

**Authors:** Steve Zimmerman

9 Jul 2026

PGPH-D-25-03800R2

Sex-Specific Correlates of Schistosoma haematobium Infection in a Rural Zambian Setting: A pilot study Focusing on Immune and Hematological Factors

PLOS Global Public Health

Dear Dr. Masenga,

Thank you for submitting your manuscript to PLOS Global Public Health. After careful consideration, we feel that it has merit but does not fully meet PLOS Global Public Health’s publication criteria as it currently stands. Therefore, we invite you to submit a revised version of the manuscript that addresses the points raised during the review process.

Reviewer 3 has raised some serious concerns regarding the reporting and analyses. I agree with the reviewer that the p values for some of the associations reported in Table 1 are clearly incorrect. For example, 22/51 men and 33/92 women have access to clean water; you would need a much larger sample for this small difference to reach statistical significance. My calculations match the reviewer's:

x2 (1) = 0.7322, p = 0.3922

Could you please revise the manuscript to carefully address the concerns raised?

Please submit your revised manuscript by Sep 07 2026 11:59PM. If you will need more time than this to complete your revisions, please reply to this message or contact the journal office at globalpubhealth@plos.org. When you're ready to submit your revision, log on to https://www.editorialmanager.com/pgph/ and select the 'Submissions Needing Revision' folder to locate your manuscript file.

Please include the following items when submitting your revised manuscript:

- A letter that responds to each point raised by the editor and reviewer(s). You should upload this letter as a separate file labeled 'Response to Reviewers'.
- A marked-up copy of your manuscript that highlights changes made to the original version. You should upload this as a separate file labeled 'Revised Manuscript with Track Changes'.
- An unmarked version of your revised paper without tracked changes. You should upload this as a separate file labeled 'Manuscript'.

Guidelines for resubmitting your figure files are available below the reviewer comments at the end of this letter.

As the corresponding author, your ORCID iD is verified in the submission system and will appear in the published article. PLOS supports the use of ORCID, and we encourage all coauthors to register for an ORCID iD and use it as well. Please encourage your coauthors to verify their ORCID iD within the submission system before final acceptance, as unverified ORCID iDs will not appear in the published article. *Only*  the individual author can complete the verification step; PLOS staff *cannot*  verify ORCID iDs on behalf of authors.

We look forward to receiving your revised manuscript.

Kind regards,

Steve Zimmerman, PhD

PLOS Staff Editor

Journal Requirements:

If the reviewer comments include a recommendation to cite specific previously published works, please review and evaluate these publications to determine whether they are relevant and should be cited. There is no requirement to cite these works unless the editor has indicated otherwise.

Additional Editor Comments (if provided):

[Note: HTML markup is below. Please do not edit.]

Reviewers' comments:

Reviewer's Responses to Questions

**Comments to the Author**

Reviewer #2: All comments have been addressed

Reviewer #3: (No Response)

**********

publication criteria?

Reviewer #2: Yes

Reviewer #3: Yes

**********

3. Has the statistical analysis been performed appropriately and rigorously?

Reviewer #2: Yes

Reviewer #3: No

**********

4. Have the authors made all data underlying the findings in their manuscript fully available (please refer to the Data Availability Statement at the start of the manuscript PDF file)?

The PLOS Data policy

Reviewer #2: Yes

Reviewer #3: Yes

**********

5. Is the manuscript presented in an intelligible fashion and written in standard English?

Reviewer #2: Yes

Reviewer #3: Yes

**********

Reviewer #2: The authors have adequately addressed all my comments and issues raised.

Reviewer #3: General comment

In the manuscript, ``Sex-Specific Correlates of Schistosoma haematobium Infection in a Rural Zambian Setting: A Pilot Study Focusing on Immune and Hematological Factors,” Masenga et al. aim to identify and compare sex-specific immune, hematological, and environmental correlates of Schistosoma haematobium infection in a rural Zambian population. The topic is interesting and potentially valuable, particularly given the importance of sex-specific analyses in schistosomiasis research. However, I have several significant concerns regarding the statistical analysis and study design. My primary concern relates to the validity of the baseline comparisons, which substantially affects confidence in the downstream analyses. At present, I believe the manuscript requires major revision, and if these concerns cannot be adequately addressed, publication may not be appropriate.

Concerns

1 Potential inconsistency in p-values reported in Table 1: In Table 1, I assume the authors used a statistical test for comparing categorical variables between male and female groups (possibly a two-proportion Z-test). I independently checked several reported p-values and found that some match the manuscript (e.g., Schistosomiasis, p = 0.4806; Have you ever been diagnosed, p = 0.6183), but many others do not. Examples include: \\

Access to clean water; Yes (my calculation: p = 0.3922) \\

Source of drinking water; Tap (my calculation: p = 0.3236)

Signs/Symptoms; Yes (my calculation: p = 0.4451) \\

Education; Not Schooled (my calculation: p = 0.6822) \\

Protective measures; Always (my calculation: p = 0.2238) \\

These discrepancies raise concerns about how the p-values were computed and reported. The authors should clearly specify which statistical tests were used for each categorical variable and verify all reported p-values in Table 1. Since Table 1 serves as the foundation for later analyses, any errors here may affect the validity of the subsequent results and conclusions. \\

In addition, if the corrected p-values are indeed such high and non-significant for all baseline characteristics, this weakens the motivation for the manuscript's central objective of investigating sex-specific differences. The study is framed around identifying meaningful differences between male and female profiles; however, if the baseline comparisons do not support notable sex-based differences, the rationale for emphasizing sex-specific analyses should be more carefully justified.

2. Unclear variable selection for multivariate analysis: It is unclear why only the variables age, access to clean water, use of protective measures, symptoms, and lymphocyte count were selected for the multivariate analysis (at least for the male subgroup). \\

Based on Table 2, multiple variables showed low p-values and significant univariate associations. The manuscript does not clearly explain the rationale for including some variables while excluding others. Were excluded variables omitted because of collinearity, redundancy, missing data, or model instability? If so, this should be explicitly stated. The authors should provide a clear variable selection strategy for the multivariate models.

3. Presentation of univariate associations in Table 2: Table 2 presents the associations between schistosomiasis status and each variable using odds ratios. While this is informative, the results would be easier to interpret with complementary visual representations. I recommend including visual summaries such as: side-by-side bar plots for categorical variables,

stacked bar plots for proportions, or

side-by-side boxplots for continuous variables. \\

Such figures would improve readability and help readers better understand the observed differences.

4. (Minor) Table organization: I recommend separating Table 1 into two tables: one for categorical variables (frequency and percentage), and

one for continuous variables (median and IQR).

5. (Minor) Lines 204–206: the authors mention the use of Mann–Whitney U tests and Chi-square tests. I recommend explicitly labeling the statistical test used for each analysis in the tables or other appropriate places.

**********

what does this mean?). If published, this will include your full peer review and any attached files.

**Do you want your identity to be public for this peer review?**  If you choose “no”, your identity will remain anonymous but your review may still be made public.

For information about this choice, including consent withdrawal, please see our Privacy Policy

Reviewer #2: No

Reviewer #3: No

**********

[NOTE: If reviewer comments were submitted as an attachment file, they will be attached to this email and accessible via the submission site. Please log into your account, locate the manuscript record, and check for the action link "View Attachments". If this link does not appear, there are no attachment files.]

# Author comment: Author response to Decision Letter 3

**DOI:** 10.1371/journal.pgph.0005692.r006

15 Sep 2026

Attachment Submitted filename: Response to Reviewers.docx

# Peer review: Decision Letter 3

**DOI:** 10.1371/journal.pgph.0005692.r007

**Authors:** Helen Howard

11 Oct 2026

PGPH-D-25-03800R3

Sex-Specific Correlates of Schistosoma haematobium Infection in a Rural Zambian Setting: A pilot study Focusing on Immune and Hematological Factors

PLOS Global Public Health

Dear Dr. Masenga,

Thank you for submitting your manuscript to PLOS Global Public Health. After careful consideration, we feel that it has merit but does not fully meet PLOS Global Public Health’s publication criteria as it currently stands. Therefore, we invite you to submit a revised version of the manuscript that addresses the points raised during the review process.

Please submit your revised manuscript by Nov 08 2026 11:59PM. If you will need more time than this to complete your revisions, please reply to this message or contact the journal office at globalpubhealth@plos.org. When you're ready to submit your revision, log on to https://www.editorialmanager.com/pgph/ and select the 'Submissions Needing Revision' folder to locate your manuscript file.

Please include the following items when submitting your revised manuscript:

- A letter that responds to each point raised by the editor and reviewer(s). You should upload this letter as a separate file labeled 'Response to Reviewers'.
- A marked-up copy of your manuscript that highlights changes made to the original version. You should upload this as a separate file labeled 'Revised Manuscript with Track Changes'.
- An unmarked version of your revised paper without tracked changes. You should upload this as a separate file labeled 'Manuscript'.

Guidelines for resubmitting your figure files are available below the reviewer comments at the end of this letter.

As the corresponding author, your ORCID iD is verified in the submission system and will appear in the published article. PLOS supports the use of ORCID, and we encourage all coauthors to register for an ORCID iD and use it as well. Please encourage your coauthors to verify their ORCID iD within the submission system before final acceptance, as unverified ORCID iDs will not appear in the published article. *Only*  the individual author can complete the verification step; PLOS staff *cannot*  verify ORCID iDs on behalf of authors.

We look forward to receiving your revised manuscript.

Kind regards,

Helen Howard

Staff Editor

PLOS Global Public Health

Journal Requirements:

If the reviewer comments include a recommendation to cite specific previously published works, please review and evaluate these publications to determine whether they are relevant and should be cited. There is no requirement to cite these works unless the editor has indicated otherwise.

Please review your reference list to ensure that it is complete and correct. If you have cited papers that have been retracted, please include the rationale for doing so in the manuscript text, or remove these references and replace them with relevant current references. Any changes to the reference list should be mentioned in the rebuttal letter that accompanies your revised manuscript. If you need to cite a retracted article, indicate the article’s retracted status in the References list and also include a citation and full reference for the retraction notice.

Additional Editor Comments (if provided):

[Note: HTML markup is below. Please do not edit.]

Reviewers' comments:

Reviewer's Responses to Questions

**Comments to the Author**

Reviewer #3: All comments have been addressed

**********

publication criteria?

Reviewer #3: Yes

**********

3. Has the statistical analysis been performed appropriately and rigorously?

Reviewer #3: Yes

**********

4. Have the authors made all data underlying the findings in their manuscript fully available (please refer to the Data Availability Statement at the start of the manuscript PDF file)?

The PLOS Data policy

Reviewer #3: Yes

**********

5. Is the manuscript presented in an intelligible fashion and written in standard English?

Reviewer #3: Yes

**********

Reviewer #3: The authors addressed all my concerns well. I still two minor concerns.

1. Text formatting in Tables 1 and 2: The table headings and p-values appear inconsistently formatted, with some text shown in black or gray and in regular or bold font. Please check whether this is a formatting issue or an intentional distinction. If the formatting is intentional, please clarify its purpose and apply it consistently throughout the tables.

2. Figures 1 and 2: Using the same scale for all measures makes the confidence intervals of many variables invisible. Consider revising the graphical format or scale to improve readability. If feasible, I would also suggest presenting the male and female results side by side in a single figure, which would facilitate direct comparison between the two groups.

**********

what does this mean?). If published, this will include your full peer review and any attached files.

**Do you want your identity to be public for this peer review?**  If you choose “no”, your identity will remain anonymous but your review may still be made public.

For information about this choice, including consent withdrawal, please see our Privacy Policy

Reviewer #3: No

**********

[NOTE: If reviewer comments were submitted as an attachment file, they will be attached to this email and accessible via the submission site. Please log into your account, locate the manuscript record, and check for the action link "View Attachments". If this link does not appear, there are no attachment files.]

# Author comment: Author response to Decision Letter 4

**DOI:** 10.1371/journal.pgph.0005692.r008

13 Oct 2026

Attachment Submitted filename: Response to Reviewers (2).docx

# Editor report: Decision Letter 4

**DOI:** 10.1371/journal.pgph.0005692.r009

**Authors:** Julia Robinson

19 Oct 2026

Sex-Specific Correlates of Schistosoma haematobium Infection in a Rural Zambian Setting: A pilot study Focusing on Immune and Hematological Factors

PGPH-D-25-03800R4

Dear Prof. Masenga,

We are pleased to inform you that your manuscript 'Sex-Specific Correlates of Schistosoma haematobium Infection in a Rural Zambian Setting: A pilot study Focusing on Immune and Hematological Factors' has been provisionally accepted for publication in PLOS Global Public Health.

Before your manuscript can be formally accepted you will need to complete some formatting changes, which you will receive in a follow up email. A member of our team will be in touch with a set of requests.

Please note that your manuscript will not be scheduled for publication until you have made the required changes, so a swift response is appreciated.

IMPORTANT: The editorial review process is now complete. PLOS will only permit corrections to spelling, formatting or significant scientific errors from this point onwards. Requests for major changes, or any which affect the scientific understanding of your work, will cause delays to the publication date of your manuscript.

If your institution or institutions have a press office, please notify them about your upcoming paper to help maximize its impact. If they'll be preparing press materials, please inform our press team as soon as possible -- no later than 48 hours after receiving the formal acceptance. Your manuscript will remain under strict press embargo until 2 pm Eastern Time on the date of publication. For more information, please contact globalpubhealth@plos.org.

Thank you again for supporting Open Access publishing; we are looking forward to publishing your work in PLOS Global Public Health.

Best regards,

Julia Robinson

Executive Editor

PLOS Global Public Health

***********************************************************

Reviewer Comments (if any, and for reference):
